# The Associations between Depression, Acculturation, and Cardiovascular Health among African Immigrants in the United States

**DOI:** 10.3390/ijerph19116658

**Published:** 2022-05-30

**Authors:** Nwakaego A. Nmezi, Ruth-Alma Turkson-Ocran, Carolyn M. Tucker, Yvonne Commodore-Mensah

**Affiliations:** 1 Division of Rehabilitation Psychology and Neuropsychology, Department of Physical Medicine and Rehabilitation, Johns Hopkins University, Baltimore, MD 21218, USA; 2 Section for Research, Division of General Medicine, Beth Israel Deaconess Medical Center, Boston, MA 02215, USA; rturkson@bidmc.harvard.edu; 3 Department of Psychology, University of Florida, Gainesville, FL 32611, USA; cmtucker@ufl.edu; 4 School of Nursing, Johns Hopkins University, Baltimore, MD 21218, USA; ycommod1@jhu.edu

**Keywords:** depression, cardiovascular health, immigrants

## Abstract

Cardiovascular disease (CVD) is the leading cause of death both globally and in the United States (U.S.). Racial health disparities in cardiovascular health (CVH) persist with non-Hispanic Black adults having a higher burden of CVD morbidity and mortality compared to other racial groups. African immigrants represent an increasingly growing sub-population of the overall U.S. non-Hispanic Black adult population, however little is known about how specific psychological and social factors (i.e., depression and acculturation) influence the CVH of U.S. African immigrants. We sought to examine the association between severity of depression symptomology and CVH among African immigrants, and whether acculturation moderated the relationship between severity of depression symptoms and CVH. Study participants were those in the African Immigrant Health Study conducted in the Baltimore-Washington D.C. area. Severity of depression symptoms were assessed using the Patient Health Questionnaire-8 (PHQ-8). CVH was assessed using the American Heart Association Life’s Simple 7 metrics and categorized as poor, intermediate, and ideal CVH. Acculturation measured as length of stay and acculturation strategy was examined as a moderator variable. Multivariable logistic regression was used to examine the association between depression and CVH and the moderating effect of acculturation adjusting for known confounders. In total 317 African immigrants participated in the study. The mean (±SD) age of study participants was 46.9 (±11.1) and a majority (60%) identified as female. Overall, 8.8% of study participants endorsed moderate-to-severe symptoms of depression. African immigrants endorsing moderate-to-severe levels of depression were less likely to have ideal CVH compared to those with minimal-to-mild symptoms of depression (Adjusted Odds Ratio [AOR]: 0.42, 95% CI: 0.17–0.99). Acculturation measured either as length of stay or acculturation strategy did not moderate the relationship between depression and CVH among study participants. Study participants exhibited elevated levels of symptoms of depression. Greater severity of depression symptoms was associated with worse CVH. Efforts to treat and prevent CVD among African immigrants should also include a focus on addressing symptoms of depression within this population.

## 1. Introduction

Cardiovascular disease (CVD) is the leading cause of death both globally and in the United States (U.S.) [1,2]. Worldwide, CVD accounts for nearly 30% of annual deaths; in the U.S., 30.4% of deaths in 2008 were attributed to CVD [1,3]. CVD is a preventable disease; however, between 2012–2013, CVD-associated health care expenditures exceeded $316.1 billion, these expenditures are expected to rise to $918 billion by 2030 [1]. Racial and ethnic differences in cardiovascular health (CVH) in the U.S. are well documented [4]. Racial and ethnic minorities, particularly non-Hispanic Black adults have higher burden of CVD morbidity and mortality compared to other racial groups [1,4,5].

Non-Hispanic Black adults in the U.S. are diverse and include U.S.-born and recently, foreign-born individuals. In 2016, 9% of the overall U.S. Black population were foreign-born, and 39% were African immigrants [6]. The proportion of African immigrants in the U.S. rose 41% between 2000 and 2013 [7]. Despite consistent evidence of poor CVH among non-Hispanic Black adults in the U.S. [8,9], evidence regarding the CVH and prevalence of CVD risk factors among African immigrants is scarce.

The vast majority of CVH research has focused on modifiable biological and behavioral risk factors for poor CVH including hypertension, diabetes, high cholesterol, and low engagement in health promoting behaviors (e.g., physical activity and healthy eating). However, there is growing evidence on the relationship between psychological factors (e.g., depression) and CVH [4,10,11,12,13,14,15]. To date, findings from several research studies, including a recent scientific statement from the American Heart Association suggest that psychological conditions such as depression are associated with poor CVH outcomes [1,13,16,17,18].

Although the relationship between CVH and depression is complex, depression has been shown to negatively impact CVH by increasing risk factors for CVD, increasing dysregulation of the sympathetic nervous system and hypothalamic-pituitary-adrenal (HPA) axis, and reducing engagement in health-promoting behaviors associated with ideal CVH [1,16]. Up to 35% of men and 65% of women who experience a cardiovascular event will also meet criteria for symptoms of depression including major depressive disorder (MDD) [16]. Furthermore, MDD is associated with increased risk of experiencing a cardiovascular event in individuals without a prior history of CVD and increased risk of poor cardiovascular outcomes following a cardiac event [19].

Findings from the 2013–2016 National Health and Nutrition Examination Survey suggests that the prevalence of depression among Black males was 7.1% and 11.0% among Black females in comparison to 5.2% and 10.5% among their White counterparts. The combined burden on CVD and depression among this group suggests that Black adults may be at increased risk for comorbid CVD and depression and associated functional impairments. In a national sample of middle-aged adults (aged 50 and older) Black adults were found to have higher odds of co-morbid CVD and depression compared to White adults (OR:2.11; 95% CI: 1.21–3.70) [20]. Additionally, Black adults with co-morbid CVD and depression were more likely to report diminished functional impairment than non-Hispanic white adults [20].

Foreign born Black adults—such as African immigrants—residing in the U.S. may possess unique risk factors for poor cardiovascular and mental health outcomes. One such risk factor is acculturation. Contemporary research suggests that acculturation is associated with a decline in CVH among African immigrants with increased length of U.S. residence [21,22]. Explanations for this phenomenon include: reduced engagement in health promoting behaviors and increased rates of obesity, hypertension, diabetes, and hyperlipidemia [21,23,24]. The role of acculturation in the relationship between depression and CVH among African immigrants remains unexplored.

African immigrants may be at increased risk for comorbid symptoms of depression and poor CVH. Research examining the prevalence of depression among African immigrants suggests symptoms of depression may be particularly high among this group [25,26]. Prior studies have documented poor CVH among African immigrants including visceral adiposity [27], hypertension and overweight/obesity [28,29]. Little is known about the degree to which symptoms of depression or acculturation are associated with CVH among African immigrants. Thus, we examined the association between severity of depression symptomology and CVH among study participants, and whether acculturation moderates the relationship between severity of depression symptoms and CVH.

## 2. Materials and Methods

### 2.1. Participants

The African Immigrant Health Study (AIHS) was a cross-sectional study conducted in the Baltimore-Washington, District of Columbia metropolitan area between June 2017 and April 2019. Individuals who met the following inclusion criteria were eligible to participate in the study: (a) self-identifying as a first-generation immigrant from one of the following African countries: Ghana, Nigeria, Liberia, Sierra Leone, and Cameroon, (b) aged 30 years or older, (c) able to communicate in English, and (d) residing in the Baltimore/Washington D.C. metro area at the time of the study. These five countries were selected because approximately 50% of African immigrants in this metropolitan area were born in West Africa [30]. The African countries included in this study are representative of the countries of origin of the African immigrant population of Maryland. A survey of African immigrants in Maryland revealed that in 2017 there were more than 150,000 residing in the state of Maryland. Ghana, Nigeria, Liberia, Sierra Leone, and Cameroon were among the 25 African countries included in this survey [31].

A convenience sample of participants were recruited from local religious and community organizations with the use of recruitment flyers, emails, oral or written announcements, and on-site recruitment sign-up at sites that agreed to participate in the study on the day of data collection. Recruiting from religious and community organization was ideal as previous research indicates that such organizations can help facilitate recruitment of populations traditionally underrepresented in research studies [32,33,34]. Eligible participants provided oral consent to participate and received a $10 gift card for providing data and completing survey questionnaires. Institutional review board (IRB) approval was obtained at the last author’s academic institution.

### 2.2. Outcome

The primary outcome variable was CVH. CVH was classified according to the American Heart Associaton (AHA) Life’s Simple 7 metric for blood pressure, diabetes, high cholesterol, smoking behavior, diet, and physical activity [35]. CVH was assessed using a questionnaire and physical measurements of height, weight, and blood pressure (BP). BP measurements were obtained using an Omron 10 Series automatic BP monitor (BP786N). A total of three BP readings were obtained, and the mean of the second and third diastolic and systolic BP readings were utilized to determine hypertension classification. Height was measured with the SECA^®^ Stadiometer, and weight was measured using the SECA^®^ Robusta 813 scale. The following information were self-reported and obtained via a researcher developed health and demographic questionnaire: health and behavioral risk factors of CVH: (1) current or past use of tobacco products (e.g., cigarettes, cigars, or pipes), (2) Body Mass Index (BMI), (3) frequency of weekly engagement in moderate or vigorous levels of physical activity (i.e., at least 150 min of moderate physical activity [e.g., brisk walking] or 75 min of vigorous physical activity [e.g., aerobic exercise]), (4) engagement in dietary behaviors (i.e., consumption of the recommended daily servings of fruits and vegetables), (5) a diagnosis of diabetes, and (6) a diagnosis of high cholesterol.

Participants were categorized as possessing either “poor”, “intermediate” or “ideal” CVH. Similar to prior research focused on examining CVH using the AHA Life’s Simple 7 metric, study participants were given 1-point for each ideal CVH metric they possessed. Participants who possessed an ideal health metric were those who met AHA guidelines for possessing ideal levels of the following health and behavioral factors which are defined as: “nonsmoking, body mass index < 25 kg/m^2^, physical activity at goal levels, and pursuit of a diet consistent with current guideline recommendations), untreated total cholesterol < 200 mg/dL, untreated blood pressure < 120/< 80 mm Hg, and fasting blood glucose < 100 mg/dL” [36]. Participants possessing 0–2 ideal CVH metrics were classified as having “poor CVH”. Participants possessing 3–4 ideal CVH metrics were classified as having “intermediate CVH”. Lastly, participants possessing 5–7 ideal CVH metrics were classified as having “ideal CVH” [10,35].

### 2.3. Independent Variable

The exposure variable was depressive symptomology assessed using the Patient Health Questionnaire (PHQ-8) [37]. The PHQ-8 is a self-report inventory designed to assess the severity of symptoms of depression within the past 2-weeks. The 8 items are rated on a 4-point Likert scale where 0 = “Not at all” and 3 = “Nearly every day.” Scores for symptom severity are categorized as minimal severity (PHQ-8 score ≤ 4); mild severity (PHQ-8 score 5–9), moderate severity (PHQ-8 score 10–14); and severe (PHQ-8 score ≥ 20). PHQ-8 scores ≥ 10 indicate clinically significant depression, have been validated, and demonstrate adequate sensitivity (88%) and specificity (88%) [37]. The Cronbach’s alpha for this sample of African immigrants was 0.85. The sum score for the PHQ-8 was used to assess severity of depression symptomology. Individuals were characterized as having minimal-to-mild depression (score range: 1 to 9) or moderate-to-severe depression (score range: 10 to ≥20).

### 2.4. Moderator Variable

We assessed acculturation as a moderator of the relationship between depression symptomology and CVH, we used a unidimensional measure of acculturation (length of stay [LOS] in the U.S.) and a bi-dimensional measure of acculturation (i.e., acculturation strategy). Consistent with previous research [28,38,39], acculturation level was dichotomized as <10 years (low acculturation) versus ≥10 years (high acculturation). A modified version of the Psychological Acculturation Scale (PAS) [40], which has been administered among African immigrants [29], was used to measure acculturation along the African and American dimensions. Items on the scale were rated on a 5-point Likert scale where 0 = “Totally disagree” and 4 = “Totally agree”. A sample item from the modified PAS is “I feel proud to be part of American culture.” Mean scores were calculated for each dimension and participants were categorized into acculturation strategies in accordance with their mean score on each dimension. The resulting acculturation strategies were as follows: (a) Integrationist (identifying equally with both African and American cultures), (b) Assimilationist (a stronger identification with American culture), (c) Traditionalist (a stronger identification with African culture), and (d) Marginalist (neither a strong identification with African nor American culture). The Cronbach’s alpha for African dimension was 0.94 and 0.85 for the American dimension.

### 2.5. Covariates

Covariate variables included age (continuous), sex assigned at birth (male, female), annual household income, level of education, health insurance status, and marital status and were assessed using the sociodemographic questionnaire.

### 2.6. Statistical Analyses

Sample size estimation was conducted to determine the appropriate sample size for the present study. The following risk factors for CVD were used to calculate the appropriate sample size needed for the present study: (a) Type II diabetes, (b) obesity, and (c) hypertension. The formula for determining sample size for cross sectional clinical studies was used to calculate sample size for the proposed study. Specifically, the formula used was: *n = z*^2^ × *p*(1 − *p*)/*d*^2^ where *z =* test statistic corresponding to a 95% confidence interval, *p =* the prevalence of the disease in the population, and *d =* the level of precision desired given the prevalence of the disease within the population [41]. A precision of 5% was selected for use since it is considered appropriate when the prevalence of a disease in the population ranges from 10–90% [41]. As a result, it was determined that at least 384 adult individuals would be needed for the study.

A total of 395 adults participated in the study. However, 78 of them were missing data on the primary outcome variable and were thus excluded from the study, resulting in an analytic sample of 317 individuals. Sociodemographic characteristics of study participants was examined using chi-square tests and analysis of variance (ANOVA) tests. Continuous variables were summarized using the mean and standard deviation whereas categorical variables were summarized using frequencies and percentages. Ordinal logistic regression was used to examine the relationship between depressive symptoms and CVH among African immigrants. Model 1 was unadjusted, Model 2 was adjusted for age and sex, and Model 3 was adjusted for age, sex, annual household income, level of education, health insurance status, and marital status. Two separate ordinal logistic regression models were run; they included the interaction between acculturation (LOS or acculturation strategy) and depressive symptoms to examine the moderating effect of acculturation on CVH. Results of these analyses were not statistically significant and thus not represented in the tables below. The results of the analyses were considered statistically significant at a two-sided alpha level of 0.05. All analyses were conducted using Statistical Package for the Social Sciences (SPSS) version 25.

## 3. Results

### 3.1. Sociodemographic Characteristics of Study Participants

We included 317 participants with mean (±SD) age of 46.9 (±11.1) years. Overall, 60% of study participants identified as female. Also, 72% were married and 64% had at least a Bachelor’s degree. Despite attaining high levels of education, only 42% reported an annual household income ≥$70,000. The majority of participants were from Ghana (32%) and from Nigeria (37%). Approximately 60% of participants reported residing in the U.S. for ≥10 years. Significant differences in CVH profile across the sociodemographic characteristics were observed. Individuals with poor CVH were more likely to be older (*p* < 0.001), have lived in the U.S. for ≥10 years (*p* < 0.001), and to identify with a Traditionalist acculturation strategy (*p* = 0.004). The results of the descriptive analyses applied to the sociodemographic data are presented in Table 1.

### 3.2. CVH Health Profile of Study Participants

CVH classifications were generated using a two-Step cluster analysis procedure which is designed to identify natural groupings among a set of data to determine group membership [42]. A total of 7 CVD risk factors (elevated BMI, diabetes diagnosis, cholesterol diagnosis, tobacco use, mean systolic blood pressure, mean diastolic blood pressure, and diet) were used to conduct the cluster analyses. Study participants were categorized in 6 clusters. Individuals in each cluster had a variance of ideal CVH health metrics ranging from a minimum of 1 ideal CVH metric (e.g., Cluster 3) to a maximum of 5 ideal CVH metrics (e.g., Cluster 5). The outcome of the cluster analysis is represented in Table 2. To develop the final CVH outcome variable individuals in Cluster 5 were coded as having ideal CVH (5 or more CVH metrics at the ideal level), individuals in Clusters 2 and 4 were coded as having intermediate CVH (3–4 ideal CVH metrics), and individuals in Clusters 1, 3 and 6 were coded as having poor CVH (0–2 ideal CVH metrics).

### 3.3. Prevalence of Depression among Study Participants

The majority (74.2%) of study participants did not report experiencing symptoms of depression. In contrast, 16.8% of study participants reported mild symptoms of depression and 8.8% reported moderate-to-severe symptoms of depression. The prevalence of depression symptomology was higher among females in comparison to males (27% to 17% respectively), although this difference was not statistically significant (*p* = 0.96).

### 3.4. Association between Depression and CVH among Study Participants

After adjusting for known confounders (age, sex, annual household income, education level, health insurance status, and marital status), there was a significant association between severity of depression symptoms and CVH (*p* = 0.05). Individuals with moderate-to-severe symptoms of depression were less likely to meet criteria for ideal CVH (adjusted OR: 0.42; 95% CI, 0.17–0.99) compared to those with minimal-to-mild symptoms of depression (Table 3).

### 3.5. Acculturation as a Moderator of the Relationship between Depression and CVH

An interaction term for length of U.S. stay (LOS), and depression was created to examine the moderating effect of LOS on the relationship between depression and CVH among study participants and was the product of LOS and depression. After adjusting for age, sex, annual household income, education level, health insurance status, and marital status LOS in the U.S. did not moderate the relationship between the severity of symptom of depression and CVH (*p* = 0.30).

Similarly, an interaction term for acculturation strategy and depression was created to examine the moderating effect of acculturation strategy on the relationship between depression and CVH. Due to the small number of individuals who identified as Assimilationists, regression analyses included only those who identified as Integrationist, Traditionalist, or Marginalist. Three separate logistic regression models were conducted to examine the moderating effect of acculturation strategy on the relationship between CVH and depression. After adjusting for known confounders (age, sex, annual household income, education level, health insurance status, and marital status), acculturation was not a moderator of the relationship between depressive symptoms and poor CVH (*p* = 0.13), intermediate CVH (*p* = 0.93), or ideal CVH (*p* = 0.71).

## 4. Discussion

Among African immigrants, we observed a significant association between depression and CVH, such that moderate-severe symptoms of depression were related to lower levels (poor, intermediate) of CVH. Consistent with national U.S. data [43], a minority of study participants met criteria for ideal CVH (i.e., 5 or more CVH metrics at the ideal level). In total, 12.7% of study participants met the criteria for ideal CVH. However, the rates of ideal CVH among study participants was slightly higher than what has been observed among U.S. Black adults. Approximately 11.8% of U.S. Black adults aged 20 or older met criteria for ideal CVH when compared to other racial and ethnic groups between 2015 and 2016 [43].

We used two measures of acculturation—length of stay (unidimensional) and acculturation strategy (bi-dimensionial). Most participants (57.7%) reported residing in the U.S. for ≥10 years and identified as being Traditionalist (41.0%). Only a minority of study participants identified as Assimilationist (4.0%). The distribution of study participants across the aforementioned acculturation strategies is not unique to the current study. A similar distribution was observed among Nigerian and Ghanaian immigrants residing in the U.S. [29]. A majority (97%) of study participants in that study also identified as either Traditionalist or Integrationist and very few individuals identified as Marginalist or Assimilationist [29].

Approximately 26% of study participants reported experiencing mild-severe symptoms of depression. Research examining prevalence of depression among foreign-born U.S Black adults is scarce. However, available epidemiological data suggests that Black adults experience symptoms of depression such as sadness and hopelessness at higher rates than non-Hispanic white adults [44]. When investigating overall prevalence of symptoms of depression among U.S. non-Hispanic Black adults, Brody and colleagues observed that prevalence of depression among U.S. non-Hispanic Black adults was 9.2% in comparison to 7.9% for non-Hispanic white adults [45]. The observed prevalence of symptoms of depression among study participants suggest that current rates of depression may be comparable to rates of depression in the aforementioned broad sample of U.S. non-Hispanic Black adults not stratified by country of birth.

As hypothesized, depressive symptoms were associated with poorer CVH. Participants who reported moderate-to-severe symptoms of depression exhibited a significantly lower odds (58% decrease) of having ideal CVH in comparison to study participants who reported minimal-to-mild symptoms of depression. In moderation analysis, length of stay or acculturation strategy did not moderate the relationship between severity of symptoms of depression and CVH. To date, research examining the association between acculturation and psychological variables known to negatively influence CVH has yielded mixed results. Some studies have reported a positive association between acculturation and mental health, others studies have reported a negative association between acculturation and mental health, and others reported a null association [46,47,48]. Furthermore, these research studies have focused on Asian and Hispanic populations.

Prior studies have shown that depression is associated with increased risk for CVD [20], poor prognosis following a cardiovascular event (e.g., heart attack, stroke) [19], and poor health behaviors [10,35]. Despite the known relationship between symptoms of depression and CVD, few studies have examined the association between symptoms of depression and CVH among foreign-born U.S. Black adults—such as African immigrants. The results of the present study are in line with available research which indicates that symptoms of depression are related to poor CVH among U.S. Black adults [49,50]. Studies exploring symptoms of depression among Black adults in sub-Saharan Africa have also found a significant relationship between symptoms of depression and diminished CVH among Black sub-Saharan Africans [51]. The results of this study suggest that additional research that explores the relationship between symptoms of depression and CVH among U.S. African immigrants is needed. The results of the present study also highlight clinical implications for African immigrants seeking treatment for CVD or recently diagnosed with CVD. In particular, African immigrants experiencing depressive symptoms may be at increased risk for the negative CVH outcomes in part due to the known negative relationship between CVH and depression. Therefore, clinicians should consider routine screening for symptoms of depression among this population and use culturally sensitive practices for discussing the relationship between CVH and depressive symptoms as well as provide appropriate resources for managing symptoms of depression.

Currently, only one study has explored how risk factors for poor CVH outcomes among U.S. immigrants from Africa may be influenced by acculturation. In their study, Commodore-Mensah and colleagues [29] observed increased risk for poor CVH (i.e., ≥3 more CVD risk factors) among Traditionalists, which align with the results of the current study. Like the majority of studies exploring health outcomes among foreign born U.S. adults, research examining the relationship between CVH and psychiatric conditions (affective disorders) has not focused on U.S. Black adult populations. Nevertheless, this research suggests that CVD risk is higher among adults with elevated rates of psychiatric disorders such as depression [52,53]. Although this research suggests that the relationship between CVH and depression may vary across different Latinx populations these researchers have not examined how acculturation may affect the relationship between depression and CVH. Additional research focused on understanding how the psychological process of acculturation may influence CVH within foreign born U.S. Black adults such as African immigrants is needed.

The present study has a few limitations that should be taken into consideration when interpreting its results. The first is the use of a cross-sectional research design which does not permit causal inferences. Future studies seeking to examine the relationships among depression, acculturation, and CVH of African immigrants from Africa may benefit from utilizing a longitudinal design. Second is the use of self-reported data for high cholesterol and diabetes. Self-report measures are susceptible to social desirability bias. Furthermore, use of self-reported data for diabetes and high cholesterol may have influenced the accuracy of the calculated AHA Life’s Simple 7 score for CVH which is typically calculated with biomedical data for high cholesterol and diabetes. Although self-reported high cholesterol tends to be less accurate than biomedical measurements of high cholesterol, self-reported diabetes tend to be highly correlated with biomedical measurements of diabetes [54,55,56,57].

Third, the use of Two-Step Cluster Analysis to develop the CVH outcome variable may have resulted in diminished power to detect a relationship between the outcome and predictor variables. This is primarily due to the loss of data that resulted from the use of this statistical procedure as observations with any missing data were dropped and thus not given a CVH categorization. Lastly, the present study utilized a convenience sampling method and only included U.S. African immigrants from the Baltimore-Washington DC metropolitan area. While convenience sampling is a common sampling methodology it is associated with sample bias and limitations in generalizability. African immigrants residing in the Baltimore-Washington DC metropolitan area may be demographically different from those who chose not to participate. 

It is possible that depression symptom severity and the cardiovascular health profile of the overall population of African immigrants in the Baltimore-Washington DC Metro area differs from that observed in the study sample. For cardiovascular health, it is possible for instance, that the prevalence of smoking may be lower among churchgoers than those who do not belong to a religious institution. For depression it is possible that people who are religious are less likely to endorse symptoms of depression due to their religious beliefs; therefore, the prevalence of depression may be underestimated. Furthermore, although a high proportion of African immigrants reside in this area, large populations of African immigrants can also be found in the southern U.S (Texas, Georgia) and New York City [20]. Therefore, the results of this study may not be generalizable to African immigrants residing in other areas of the U.S.

This study is unique and has several noteworthy strengths. To date, this is the first known study that has examined the influence of depression on CVH among a sample of U.S. African immigrants. Rates of depression within this group of African immigrants was similar to rates of depression found among U.S. Black adults in general. This study makes a meaningful contribution to the available body of health psychology literature that focuses on examining the relationship between psychological variables such as depression and physical health outcomes such as CVH.

Another strength of the present study is the focus on within group differences of a underserved population. U.S. Black adults are disproportionately impacted by CVD, however the vast majority of research focused on addressing CVD health disparities among U.S. Black adults has tended to group all Black adults together. Unfortunately, this practice has resulted in the loss of meaningful information that may help researchers develop interventions that target both broad and specific factors (e.g., physical activity and acculturation) that may contribute to CVD health disparities across the U.S. Black adult diaspora. For example, within the group of U.S. African immigrant’s acculturation was positively associated with worse CVH. This is especially important and indicates that behavioral health interventions, specifically those targeting improved CVH among U.S. Black adults should also consider the ethnic diversity within the U.S. Black adult population and the potential influence of acculturation on CVH outcomes.

Although the present study utilized self-report data for diabetes and high cholesterol, physical measurements for hypertension were obtained for study participants. Doing so increased the accuracy of the calculated AHA Life’s Simple 7 score and also provided a more reliable estimate of the prevalence of hypertension among study participants.

Lastly, the PHQ-8—designed to measure severity of depression symptoms and the PAS—designed to measure psychological components of acculturation, showed good psychometric properties within the present study (based on Cronbach’s alpha). Therefore, demonstrating the validity of these instruments within this population and provides support for continued use of these questionnaires and inventories in future research studies with U.S. African immigrants.

## 5. Conclusions

The results of the current study highlight elevated symptoms of depression within the study participants and an association between depression symptom severity and declines in CVH. Given that depression is associated with an increased risk of CVD and is often co-morbid among adults with CVD [20], the study findings suggest that efforts to treat and prevent CVD among African immigrants should also include a focus on addressing symptoms of depression within this population. Furthermore, practicing clinicians should consider the use of culturally sensitive inventories or questions when assessing depression symptomology to help increase the likelihood of appropriate assessment of psychological factors known to impact CVH which may consequently result in improved CVH among African immigrants.

Our study suggests that findings from research on U.S. Blacks that predominantly include individuals that identify as African American may not necessarily generalize to African immigrants in the U.S. The language, foods, and cultural traditions unique to African immigrants suggest that the influences on their health are likely unique and thus should be studied. The present study provides the impetus for more research on the CVH of African immigrants living in the U.S. This study provides strong support for developing and testing culturally sensitive mental health interventions in African immigrants and should be prioritized in research. More research (including qualitative research methodologies) among African immigrants are also needed to understand the contributing factors to depression among African immigrants in the U.S. and identify acculturation-related factors that may influence depression and its relationship to CVH and CVD.

## Figures and Tables

**Table 1:** Sociodemographic Characteristics of Study Participants by CVH status, *n* = 317.

Characteristic [Mean (SD) or N (%)]	Total	Poor 92 (29.0%)	Intermediate 175 (55.2%)	Ideal 50 (15.8%)	*p* Value
Age [Mean (SD)]	46.9 (11.07)	51.9 (11.1)	45.3 (10.7)	43.0 (9.2)	<0.001
Sex					0.35
Female	190 (59.9%)	51 (55.4%)	105 (60.0%)	34 (68.0%)	
Male	127 (40.1%)	41 (44.6%)	70 (40.0%)	16 (32.0%)	
Marital Status					0.16
Not Married	89 (28.4%)	33 (35.9%)	43 (24.9%)	13 (27.1%)	
Married/Living with a Partner	224 (71.6%)	59 (64.1%)	130 (75.1%)	35 (72.9%)	
Education Level					0.09
<Bachelor’s Degree	113 (36.2%)	39 (42.4%)	63 (36.4%)	11 (23.4%)	
≥Bachelor’s Degree	199 (63.8%))	53 (57.6%)	110 (63.6%)	36 (76.6%)	
Annual Household Income					0.47
<$30,000	57 (22.3%)	13 (16.9%)	35 (25.2%)	9 (22.5%)	
≥$30,000–<$70,000	91 (35.5%)	31 (40.3%)	49 (35.3%)	11 (27.5%)	
≥$70,000	108 (42.2%)	33 (42.9%)	55 (39.6%)	20 (50.0%)	
Length of Stay					<0.001
<10 Years	113 (37.4%)	16 (18.8%)	78 (45.9%)	19 (40.4%)	
≥10 Years	189 (62.6%)	69 (81.2%)	92 (54.1%)	28 (59.6%)	
Acculturation Strategy					<0.01
Integrationist	86 (27.7%)	19 (21.3%)	45 (26.0%)	22 (45.8%)	
Assimilationist	12 (3.9%)	0	11 (6.4%)	1 (2.1%)	
Traditionalist	137 (44.2%)	49 (55.1%)	71 (41.0%)	17 (35.4%)	
Marginalist	75 (24.2%)	21 (23.6%)	46 (26.6%)	8 (16.7%)	

**Table 2:** Outcome of Two-Step Cluster Analysis.

Cluster 1 *n* = 44	Cluster 2 *n* = 74	Cluster 3 *n* = 37	Cluster 4 *n* = 57	Cluster 5 *n* = 50	Cluster 6 *n* = 55
Diabetes: ideal	Diabetes: ideal	Diabetes: poor	Diabetes: ideal	Diabetes: ideal	Diabetes: ideal
Cholesterol: ideal	Cholesterol: ideal	Cholesterol: poor	Cholesterol: ideal	Cholesterol: ideal	Cholesterol: poor
Blood pressure: poor	Blood pressure: intermediate	Blood pressure: intermediate	Blood pressure: ideal	Blood pressure: ideal	Blood pressure: intermediate
Diet: intermediate	Diet: poor	Diet: intermediate	Diet: poor	Diet: ideal	Diet: intermediate
BMI: poor	BMI: poor	BMI: poor	BMI: intermediate	BMI: intermediate	BMI: poor
Tobacco: ideal	Tobacco: ideal	Tobacco: ideal	Tobacco: ideal	Tobacco: ideal	Tobacco: ideal

Note: red shading represents poor CVH; yellow shading represents intermediate CVH; green shading represents ideal CVH

**Table 3:** Ordinal Logistic Regression analysis results examining the association between severity of depression symptoms and CVH, *n* = 247.

	Unadjusted	Model 1	Model 2
Variable	OR (95% CI)	AOR (95% CI)	AOR (95% CI)
Depression			
Moderate-to-severe depression	**0.45** (**0.22–0.94**)	**0.45** (**0.21–0.96**)	**0.42** (**0.17–0.99**)
Minimal-to-mild depression	Ref	---	---
Age	---	0.93 (0.91–0.95)	0.93 (0.91–0.96)
Sex			
Female	---	1.61 (1.04–2.50)	1.64 (0.99–2.70)
Male	Ref	---	---
Annual Household Income			
≤$30,000	Ref	---	---
>$30,000–≤$70,000	---	---	0.94 (0.46–1.95)
>$70,000	---	---	1.03 (0.51–2.07)
Education Level			
<Bachelor’s Degree	Ref	---	---
≥Bachelor’s Degree	---	---	1.87 (1.05–3.34)
Health Insurance Status			
No	Ref	---	---
Yes	---	---	0.57 (0.32–1.00)
Marital Status			
Not Married	Ref	---	---
Married/Living with a Partner	---	---	1.05 (0.58–1.89)

Model 1: xxxxxx. Model 2: Adjusted for age, sex, annual household income, education level, health insurance status, and marital status. **Bold**, statistically significant at *p* ≤ 0.05. OR: Odds Ratio; AOR: Adjusted Odds Ratio; 95% CI: 95% Confidence interval.

## Data Availability

Raw data used in this study can be made available from the Principal Investigator upon request.

## References

[1] Tsao C.W., Aday A.W., Almarzooq Z.I., Alonso A., Beaton A.Z., Bittencourt M.S., Boehme A.K., Buxton A.E., Carson A.P., Commodore-Mensah Y. (2022). Heart Disease and Stroke Statistics-2022 Update: A Report From the American Heart Association. Circulation.

[2] WHO (2017). Cardiovascular Diseases (CVDs).

[3] Deaton C., Froelicher E.S., Wu L.H., Ho C., Shishani K., Jaarsma T. (2011). The global burden of cardiovascular disease. J. Cardiovasc. Nurs..

[4] Havranek E.P., Mujahid M.S., Barr D.A., Blair I.V., Cohen M.S., Cruz-Flores S., Davey-Smith G., Dennison-Himmelfarb C.R., Lauer M.S., Lockwood D.W. (2015). Social determinants of risk and outcomes for cardiovascular disease: A scientific statement from the American Heart Association. Circulation.

[5] Davis A.M., Vinci L.M., Okwuosa T.M., Chase A.R., Huang E.S. (2007). Cardiovascular health disparities: A systematic review of health care interventions. Med. Care Res. Rev..

[6] Anderson M., Lopez G. Key Facts about Black Immigrants in the U.S., 2018. Pew Research Center. http://www.pewresearch.org/fact-tank/2018/01/24/key-facts-about-black-immigrants-in-the-u-s.

[7] Anderson M. African Immigrant Population in U.S. Steadily Climbs, 2017. Pew Research Center. https://www.pewresearch.org/fact-tank/2017/02/14/african-immigrant-population-in-u-s-steadily-climbs/.

[8] Mouton C.P., Hayden M., Southerland J.H. (2017). Cardiovascular Health Disparities in Underserved Populations. Prim. Care-Clin. Off. Pract..

[9] Carnethon M.R., Pu J., Howard G., Albert M.A., Anderson C.A.M., Bertoni A.G., Mujahid M.S., Palaniappan L., Taylor H.A., Willis M. (2017). Cardiovascular Health in African Americans: A Scientific Statement from the American Heart Association. Circulation.

[10] Gaye B., Prugger C., Boutouyrie P., Thomas F., Guibout C., Perrier M.C., Pannier B., Jouven X., Empana J.P. (2015). High level of depressive symptoms as a barrier to reach an ideal cardiovascular health. The Paris Prospective Study III. Circulation.

[11] Mathews L., Ogunmoroti O., Nasir K., Blumenthal R.S., Utuama O.A., Rouseff M., Das S., Veledar E., Feldman T., Agatston A. (2018). Psychological Factors and Their Association with Ideal Cardiovascular Health Among Women and Men. J. Women’s Health.

[12] Li Z., Yang X., Wang A., Qiu J., Wang W., Song Q., Wang X. (2015). Association between Ideal Cardiovascular Health Metrics and Depression in Chinese Population: A Cross-Sectional Study. Sci. Rep..

[13] Bomhof-Roordink H., Seldenrijk A., van Hout H.P.J., van Marwijk H.W.J., Diamant M., Penninx B.W.J.H. (2015). Associations between life stress and subclinical cardiovascular disease are partly mediated by depressive and anxiety symptoms. J. Psychosom. Res..

[14] Cohen B.E., Edmondson D., Kronish I.M. (2015). State of the art review: Depression, stress, anxiety, and cardiovascular disease. Am. J. Hypertens..

[15] Kivimäki M., Steptoe A. (2018). Effects of stress on the development and progression of cardiovascular disease. Nat. Rev. Cardiol..

[16] Dhar A.K., Barton D.A. (2016). Depression and the link with cardiovascular disease. Front. Psychiatry.

[17] Penninx B.W.J.H. (2017). Depression and cardiovascular disease: Epidemiological evidence on their linking mechanisms. Neurosci. Biobehav. Rev..

[18] Levine G.N., Cohen B.E., Commodore-Mensah Y., Fleury J., Huffman J.C., Khalid U., Labarthe D.R., Lavretsky H., Michos E.D., Spatz E.S. (2021). Psychological Health, Well-Being, and the Mind-Heart-Body Connection Circulation: A Scientific Statement from the American Heart Association. Circulation.

[19] Hare D.L., Toukhsati S., Johansson P., Jaarsma T. (2014). Depression and cardiovascular disease: A clinical review. Eur. Heart J..

[20] González H.M., Tarraf W. (2013). Comorbid cardiovascular disease and major depression among ethnic and racial groups in the United States. Int. Psychogeriatr..

[21] Commodore-Mensah Y., Ukonu N., Obisesan O., Aboagye J.K., Agyemang C., Reilly C.M., Dunbar S.B., Okosun I.S. (2016). Length of Residence in the United States is Associated with a Higher Prevalence of Cardiometabolic Risk Factors in Immigrants: A Contemporary Analysis of the National Health Interview Survey. J. Am. Heart Assoc..

[22] Hamilton T.G., Palermo T., Green T.L. (2015). Health Assimilation among Hispanic Immigrants in the United States. J. Health Soc. Behav..

[23] Delavari M., Sønderlund A.L., Swinburn B., Mellor D., Renzaho A. (2013). Acculturation and obesity among migrant populations in high income countries—A systematic review. BMC Public Health.

[24] Wong S.S., Dixon L.B., Gilbride J.A., Kwan T.W., Stein R.A. (2013). Measures of acculturation are associated with cardiovascular disease risk factors, dietary intakes, and physical activity in older Chinese Americans in New York City. J. Immigr. Minor. Health.

[25] Pannetier J., Lert F., Roustide M.J., du Loû A.D. (2017). Mental health of sub-saharan african migrants: The gendered role of migration paths and transnational ties. SSM-Popul. Health.

[26] Escamilla S., Saasa S. (2020). Gendered Patterns in Depression and Anxiety among African Immigrants in the United States. J. Evid.-Based Soc. Work.

[27] O’Connor M.Y., Thoreson C.K., Ricks M., Courville A.B., Thomas F., Yao J., Katzmarzyk P., Sumner A.E. (2014). Worse Cardiometabolic Health in African Immigrant Men than African American Men: Reconsideration of the Healthy Immigrant Effect. Metab. Syndr. Relat. Disord..

[28] Commodore-Mensah Y., Samuel L.J., Himmelfarb C.D., Agyemang C. (2014). Hypertension and overweight/obesity in Ghanaians and Nigerians living in West Africa and industrialized countries. J. Hypertens..

[29] Commodore-Mensah Y., Ukonu N., Cooper L.A., Agyemang C., Himmelfarb C.D. (2017). The Association Between Acculturation and Cardiovascular Disease Risk in Ghanaian and Nigerian-Born African Immigrants in the United States: The Afro-Cardiac Study. J. Immigr. Minor. Health.

[30] State Demographics Data. https://www.migrationpolicy.org/data/state-profiles/state/demographics/MD.

[31] Geaorge Mason University Institute for Immigration Research African Immigrants in Maryland; pp. 1–3. https://d101vc9winf8ln.cloudfront.net/documents/33451/original/Africans_Maryland_Oport.pdf?1575469884.

[32] Hughson J.-A., Woodward-Kron R., Parker A., Hajek J., Bresin A., Knoch U., Phan T., Story D. (2016). A review of approaches to improve participation of culturally and linguistically diverse populations in clinical trials. Trials.

[33] Yancey A.K., Ortega A.N., Kumanyika S.K. (2006). Effective recruitment and retention of minority research participants. Annu. Rev. Public Health.

[34] Commodore-Mensah Y., Turkson-Ocran R.-A., Nmezi N.A., Nkimbeng M., Cudjoe J., Mensah D.S., York S., Mossburg S., Patel N., Adu E. (2019). Commentary: Engaging African immigrants in research—Experiences and lessons from the field. Ethn. Dis..

[35] Lloyd-Jones D.M., Hong Y., Labarthe D., Mozaffarian D., Appel L.J., Van Horn L., Greenlund K., Daniels S., Nichol G., Tomaselli G.F. (2010). Defining and setting national goals for cardiovascular health promotion and disease reduction: The american heart association’s strategic impact goal through 2020 and beyond. Circulation.

[36] España-Romero V., Artero E.G., Lee D.-C., Sui X., Baruth M., Ruiz J.R., Pate R.R., Blair S.N. (2013). A Prospective Study of Ideal Cardiovascular Health and Depressive Symptoms. Psychosomatics.

[37] Kroenke K., Spitzer R.L. (2002). The PHQ-9: A New Depression Diagnostic and Severity Measure. Psychiatr. Ann..

[38] Goel M.S., McCarthy E.P., Phillips R.S., Wee C.C. (2004). Obesity among US immigrant subgroups by duration of residence. J. Am. Med. Assoc..

[39] Kandula N.R., Diez-Roux A.V., Chan C., Daviglus M.L., Jackson S.A., Ni H., Schreiner P.J. (2008). Association of acculturation levels and prevalence of diabetes in the multi-ethnic study of atherosclerosis (MESA). Diabetes Care.

[40] Tropp L.R., Coll C.G., Alarcón O., College W., García H.A.V. (1999). Psychological acculturation: Development of a new measure for Puerto Ricans on the U.S. mainland. J. Educ. Psychol. Meas..

[41] Pourhoseingholi M.A., Vahedi M., Rahimzadeh M. (2013). Sample size calculation in medical studies. Gastroenterol. Hepatol. Bed Bench.

[42] Benassi M., Garofalo S., Ambrosini F., Sant’Angelo R.P., Raggini R., De Paoli G., Ravani C., Giovagnoli S., Orsoni M., Piraccini G. (2020). Using Two-Step Cluster Analysis and Latent Class Cluster Analysis to Classify the Cognitive Heterogeneity of Cross-Diagnostic Psychiatric Inpatients. Front. Psychol..

[43] Virani S.S., Alonso A., Benjamin E.J., Bittencourt M.S., Callaway C.F., Carson A.P., Chamberlain A.M., Chang A.R., Cheng S., Delling F.N. (2020). Heart Disease and Stroke Statistics—2020 Update: A Report From the American Heart Association. Circulation.

[44] Centers for Disease Control and Prevention Summary of Health Statistics:National Health Interview Survey, 2018. Table A-7, 2019; pp. 1–12. https://ftp.cdc.gov/pub/Health_Statistics/NCHS/NHIS/SHS/2018_SHS_Table_A-7.pdf.

[45] Brody D.J., Pratt L.A., Hughes J.P. (2018). Prevalence of Depression Among Adults Aged 20 and Over: United States, 2013–2016 Key Findings Data from the National Health and Nutrition Examination Survey. NCHS Data Brief.

[46] Allen C.D., McNealy C.A. (2015). Self-rated health in disparities research: Construct validity of SRH across racial/ethnic groups and immigrant generations among U.S. adolescents and young adults. J. Adolesc. Health.

[47] Koneru V.K., de Mamani A.G.W., Flynn P.M., Betancourt H. (2007). Acculturation and mental health: Current findings and recommendations for future research. Appl. Prev. Psychol..

[48] Yoon E., Hacker J., Hewitt A., Abrams M., Cleary S. (2012). Social connectedness, discrimination, and social status as mediators of acculturation/enculturation and well-being. J. Couns. Psychol..

[49] O’Brien E.C., Greiner M.A., Sims M., Hardy N.C., Wang W., Shahar E., Hernandez A.F., Curtis L.H. (2015). Depressive Symptoms and Risk of Cardiovascular Events in Blacks Findings From the Jackson Heart Study. Circ. Cardiovasc. Qual. Outcomes.

[50] Lewis T.T., Guo H., Lunos S., de Leon C.F.M., Skarupski K.A., Evans D.A., Everson-Rose S.A. (2011). Depressive Symptoms and Cardiovascular Mortality in Older Black and White Adults Evidence for a Differential Association by Race. Circ. Cardiovasc. Qual. Outcomes.

[51] Hamer M., Malan N.T., Harvey B.H., Malan L. (2011). Depressive symptoms and sub-clinical atherosclerosis in Africans: Role of metabolic syndrome, inflammation and sympathoadrenal function. Physiol. Behav..

[52] Cabassa L.J., Lewis-Fernández R., Wang S., Blanco C. (2017). Cardiovascular disease and psychiatric disorders among Latinos in the United States. Soc. Psychiatry Psychiatr. Epidemiol..

[53] Castañeda S.F., Buelna C., Giacinto R.E., Gallo L.C., Sotres-Alvarez D., Gonzalez P., Fortmann A.L., Wassertheil-Smoller S., Gellman M.D., Giachello A.L. (2016). Cardiovascular Disease Risk Factors and Psychological Distress among Hispanics/Latinos: The Hispanic Community Health Study/Study of Latinos (HCHS/SOL). Prev. Med..

[54] Chun H., Kim I.H., Min K.D. (2016). Accuracy of Self-Reported Hypertension, Diabetes, and Hypercholesterolemia: Analysis of a Representative Sample of Korean Older Adults. Osong Public Health Res. Perspect..

[55] Huerta J.M., Tormo M.J., Egea-Caparrós J.M., Ortolá-Devesa J.B., Navarro C. (2009). Accuracy of Self-Reported Diabetes, Hypertension, and Hyperlipidemia in the Adult Spanish Population. DINO Study Findings. Rev. Esp. Cardiol. (Engl. Ed.).

[56] Peterson K.L., Jacobs J.P., Allender S., Alston L.V., Nichols M. (2016). Characterising the extent of misreporting of high blood pressure, high cholesterol, and diabetes using the Australian Health Survey. BMC Public Health.

[57] Chrisman M., Chow W.-H., Daniel C.R., Wu X., Zhao H. (2017). Associations between language acculturation, age of immigration, and obesity in the Mexican American Mano A Mano cohort. Obes. Res. Clin. Pract..

